# Effectiveness and Toxicity of Cemiplimab Therapy for Advanced Cutaneous Squamous Cell Skin Cancer in a Community Oncology Practice

**DOI:** 10.3390/cancers17050823

**Published:** 2025-02-27

**Authors:** Tina Fung, Wolfram Samlowski, Raul Meoz

**Affiliations:** 1 Kirk Kerkorian School of Medicine, University of Nevada, Las Vegas (UNLV), Las Vegas, NV 89102, USA; fungt1@unlv.nevada.edu; 2 Comprehensive Cancer Centers of Nevada, Las Vegas, NV 89148, USA

**Keywords:** keratinocyte carcinoma, squamous cell skin cancer, checkpoint inhibitor

## Abstract

Background: The immune checkpoint inhibitor cemiplimab has significant clinical activity in unresectable and metastatic cutaneous squamous cell carcinomas. There are limited real-world data available to assess the outcome of cemiplimab treatment in patients in a community practice setting. Methods: We conducted a retrospective analysis of treatment outcomes following cemiplimab treatment (350 mg IV every 3 weeks) of squamous cell skin cancer. An exploratory analysis was performed to evaluate patient subsets, including patients with locally advanced disease, regional or distant metastases, and “too numerous to count” primaries. Another small group of patients who did not respond to the initial four doses of cemiplimab were evaluated following added radiotherapy. Results: Of the 36 patients treated, 22 (61.1%) achieved complete remission, 10 (27.8%) experienced a partial response, 3 (8.3%) had stable disease, and 1 (2.8%) developed progressive disease. The median progression-free survival for the entire cohort was over 33 months. Overall, cemiplimab was well-tolerated, with no hospitalizations due to treatment-related toxicity. Conclusions: Cemiplimab produced complete remissions in over 60% of patients with locally advanced and metastatic squamous cell skin cancers, allowing elective treatment discontinuation. Addition of radiotherapy in cemiplimab-refractory patients appeared to increase tumor responsiveness. In contrast, patients with TNTC primary tumors frequently develop new primary skin cancers. Thus, improved treatment options for this patient subset are still needed.

## Simple Summary

Some squamous cell skin cancers cannot be managed by surgery or radiation treatments. Cancer immunotherapy has proven useful for these patients. We evaluated our experience using the PD-1 antibody cemiplimab in a community practice setting. We found a high response rate in patients with locally advanced cancers, as well as those with metastases or innumerable primary cancers. Many of these patients were able to achieve a complete remission. Most of the responding patients were able to successfully stop treatment without a recurrence of their cancer. In patients suffering from innumerable skin cancers, the development of new tumors continues to be an issue requiring the development of additional treatment options.

## 1. Introduction

Cutaneous squamous cell carcinoma (CSCC) is the second most common skin cancer in the United States, after basal cell carcinoma. The precise incidence of CSCC is not easily determined, as this cancer is not reported via the NCI Surveillance, Epidemiology, and End Results (SEER) cancer registry. It has been estimated that there are more than 1,000,000 individuals who develop CSCC every year in the United States [1]. Most CSCCs are small, localized lesions that are easily treated with surgery, radiotherapy, or other ablative procedures [1]. It has more recently become apparent that occasionally patients develop more advanced stage lesions. It is estimated that 3–7% of CSCC patients develop deeply invasive local disease, or even regional and distant metastases [2,3,4,5,6,7]. For example, in 2012 it was estimated that about 5604–12,572 patients developed metastatic CSCC in the United States, and 3932–8791 died as a consequence [3].

The population most susceptible to deeply invasive or metastatic CSCC predominantly comprises elderly, fair-skinned Caucasians with a history of extensive sun exposure, particularly involving the head, neck, and upper torso regions [8]. Several tumor-related factors contribute to an increased risk of locally advanced or metastatic CSCC, including primary tumor localization to head and neck sites, indistinct and infiltrative lesion borders, rapid tumor growth, a diameter exceeding 2 cm, and invasion to a depth greater than 2.0 mm [8,9,10]. Furthermore, the presence of perineural extension increases the likelihood of aggressive disease progression [11]. Host factors, such as immunosuppression following solid organ transplantation or in patients with co-existing immune defects, such as HIV, chronic lymphocytic leukemia (CLL), and indolent lymphomas, increase the aggressiveness and metastatic potential of CSCC. Additionally, tumor recurrence following prior surgical excision or radiotherapy increases the risk for deeper invasion or metastasis [12]. Staging systems, including those from the American Joint Committee on Cancer (AJCC), the Union for International Cancer Control (UICC), and the Brigham and Women’s Hospital classification, have been used to identify high-risk patients for more intensive therapy [13,14,15].

There have been recent advances in the medical treatment of locally advanced or metastatic CSCC. Immune checkpoint inhibitors such as the PD-1 antibodies cemiplimab and pembrolizumab have shown significant clinical activity in the treatment of locally advanced or metastatic CSCC [16,17]. It should be noted that these studies included patients who had progressed after prior surgery or radiotherapy [18]. In clinical trials with these agents, rapid and deep objective responses were seen, including many patients with durable responses or remissions.

Despite these promising results, there are limited “real world” data to evaluate the effectiveness of PD-L1 inhibitors, such as cemiplimab, in advanced CSCC patients. We performed a retrospective analysis of the outcome of cemiplimab therapy for patients with advanced CSCC in a large skin cancer-focused community practice. We performed an exploratory analysis of treatment outcomes in several clinically important subsets of patients, including those with locally advanced, metastatic, and “too numerous to count” (TNTC) small primary lesions. We also describe a small group of patients treated with cemiplimab in combination with concurrent radiotherapy, who had initially failed to respond to 2–4 cycles of cemiplimab treatment.

## 2. Materials and Methods

All patients who had been treated with cemiplimab by a single oncologist (WS) were identified via a search of the iKnowMed Database (IKM-G2, McKesson, Woodlands, TX, USA). Each patient’s medical record was individually accessed, and relevant data were extracted into a password-protected spreadsheet (Microsoft Excel v16.86, Redmond, WA, USA). Patients were included in the current analysis if they were diagnosed with cutaneous squamous cell carcinoma (CSCC) and had received more than 1 dose of cemiplimab. Each patient was assigned an arbitrary unique patient number (UPN). Patients with mixed squamous and basal cell carcinoma or who had received cemiplimab treatment for other indications (i.e., basal cell carcinoma or lung cancer) were excluded.

Patient characteristics recorded in the spreadsheet included age at the start of therapy, gender, race, comorbidities, and any cause for immunosuppression. Tumor characteristics, such as the primary tumor site and prior treatments (surgery, radiotherapy, or other therapy), were recorded. Whether patients had been treated for locally advanced tumors, multifocal CSCC, or metastatic disease was noted. The sites of metastases were also recorded. Following data collection, the spreadsheet was deidentified. This study design has been reviewed by the chair of the Western IRB and has been deemed exempt from full IRB review.

### 2.1. Treatment Regimens

All patients were treated with a fixed dose of cemiplimab 350 mg IV every 3 weeks. The date of treatment initiation, duration of treatment, number of doses, and any cemiplimab-related toxicity were recorded. Occasional patients had treatment interruptions (i.e., insurance changes, comorbid illnesses, temporarily lost to follow-up). In these patients, treatment was analyzed from the time of treatment restart.

Patients who received concurrent radiotherapy in conjunction with cemiplimab administration were analyzed separately. All patients were treated using customized planning with immobilization devices. Three patients were treated using 6 MeV electrons with custom blocking and the use of bolus. Two patients were treated with intensity-modulated radiotherapy (IMRT) techniques using 6 Mev photons with a bolus as needed. The latter patients were treated with conventional fractionation with 5 treatments per week. Data collected related to these patients included the dose of radiation (cGy), number of fractions, and elapsed time (days). Interruptions of RT for toxicity were also recorded.

### 2.2. Response Assessment

Whenever possible, quantitative response assessment was used to evaluate the objective response to cemiplimab treatment. Lesions were measured using computerized tomography (CT) scans, magnetic resonance imaging (MRI), or positron emission tomography (PET) CT scans. Response was assessed by RECIST 1.1 criteria [19]. A complete response (CR) was defined as complete resolution of all lesions. A partial response (PR) was defined as a >30% reduction in the sum of cross-sectional measurements of index lesions. Progressive disease (PD) was present if there was a greater than 20% increase in the sum of bidimensional tumor measurements or new metastatic lesions developed. Stable disease (SD) was defined as tumor measurements not meeting the criteria for CR, PR, or PD.

Some patients with large, locally advanced cutaneous lesions were not measurable based on radiographic imaging. These patients were assessed in a semi-quantitative fashion by skin examinations. In these patients, disappearance of raised tumor margins and healing of ulcers indicated a complete response. This was generally confirmed by biopsies of the residual abnormal scar tissue at the prior tumor site. Partial response was indicated by the presence of residual tumor, which had decreased in size by >30%. Progressive disease was defined as either growth of the original lesion, or development of new metastatic lesions. Development of new CSCC skin primaries at remote sites was separately noted. This was not characterized as progression except in patients who were being treated for multifocal skin cancers, which were usually too numerous to count (TNTC). Local recurrence, distant metastases sites, and the development of any new malignancies (both skin and non-cutaneous malignancies) were recorded. Data collection ended on 8 June 2024.

### 2.3. Statistical Analysis

Simple descriptive statistics were calculated via the Excel spreadsheet. A Kaplan–Meier analysis was performed to evaluate progression-free survival (PFS) [20]. PFS was calculated for induction therapy from the beginning of initial CKI treatment until the date of relapse or disease progression.

## 3. Results

### 3.1. Characteristics of the Study Population

We identified 36 patients who were treated with cemiplimab for locally advanced or metastatic CSCC in our clinic (Supplemental Table S1). Our study population (26 men and 10 women) had a median age of 76.9 ± 10.6 years. There were 35 Caucasian patients and 1 Hispanic individual. Immunosuppression was suspected to be a contributory factor for aggressive CSCC tumor growth in six patients. Causes of potential immunosuppression included CLL (1), indolent non-Hodgkin’s lymphoma (NHL) (2), treated chronic hepatitis C (1), HIV (1), and treatment with azathioprine plus ustekinumab for colitis (1).

A total of 29 patients had undergone a previous surgical resection and had progressed, while 7 patients had no prior surgery. The seven patients with no prior surgery either presented with evidence of metastatic disease or presented with surgically unresectable CSCC. Twenty-six patients had not received prior radiotherapy to the current tumor site. Four patients had received other forms of local therapy.

### 3.2. Overall Outcome of Cemiplimab Treatment

**Figure 1:**
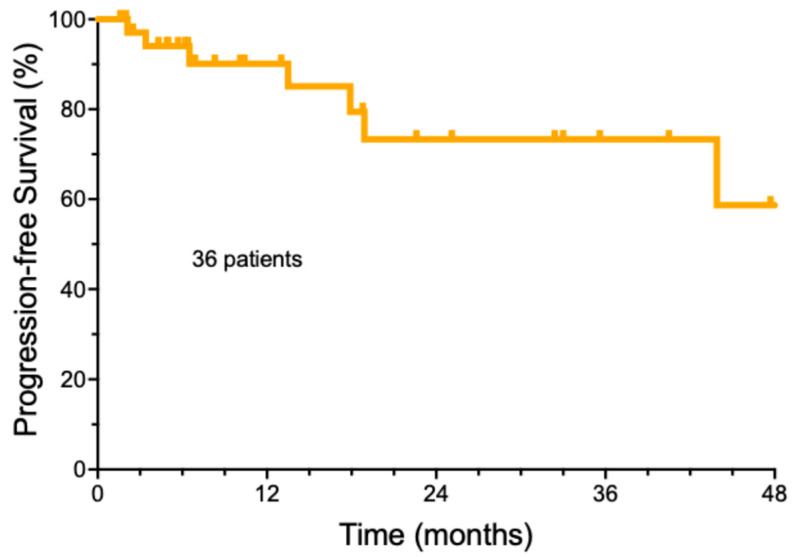
Overall progression-free survival of cemiplimab-treated cutaneous squamous cell carcinoma patients.

The follow-up of the entire group of cemiplimab-treated patients was a median of 32.9 months ± 18.4, with a range from 3.7 to 61.1 months (Supplemental Table S2). The median number of cemiplimab doses was 8 (±6.3 doses StDev, range 2–39). The median duration of cemiplimab treatment was only 5.2 months (±5.6 months). A total of 22/36 (61.1%) patients achieved a complete remission, while ten patients had a partial response (27.8%), three patients had stable disease (8.3%), and one patient developed progressive disease (2.8%). For the entire group, the estimated median progression-free survival was undefined (Figure 1). A total of 21 patients (58.3%) remained free of recurrence over the entire period of observation. The majority of the patients who achieved a remission (19/21, 90.5%) were able to electively discontinue therapy, based on a previously published strategy [21]. Another 12 patients (33.3%) were alive with persistent CSCC. Deaths due to progressive CSCC were rare. Only one patient (2.8%) died due to CSCC within the study period. It should be noted that in this elderly population, two additional patients (5.6%) died of non-cancer-related causes. One patient is currently still undergoing cemiplimab treatment, and one patient was lost to follow-up.

**Figure 2:**
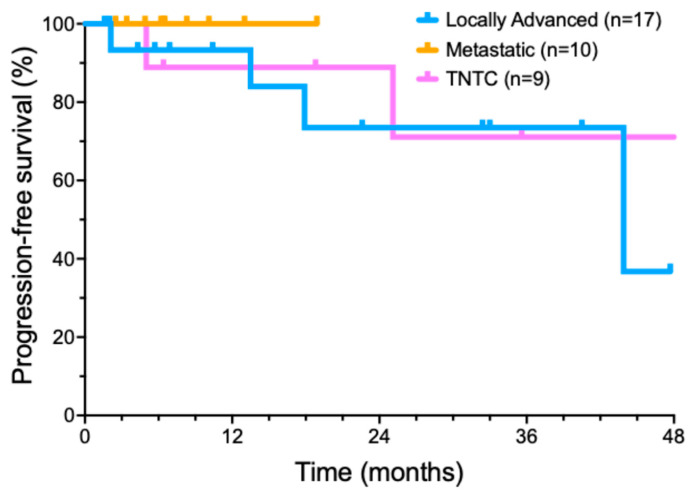
Progression-free survival of cemiplimab-treated cutaneous squamous cell carcinoma patient subsets.

We also performed an exploratory analysis of several important recurring clinical scenarios for CSCC patients (Figure 2). Nine patients presented with too many lesions to treat surgically (usually dozens or hundreds) that could no longer be managed by standard treatments. Ten patients presented with metastatic disease at diagnosis, involving a total of thirteen metastatic sites. Six of these patients had metastases from a head and neck CSCC primary to intra-parotid lymphoid tissue. In another subgroup of five patients, concurrent radiotherapy was added to locoregionally advanced disease after neoadjuvant cemiplimab cytoreductive therapy failed to produce a clinical response.

### 3.3. Cemiplimab for Treatment of Locally Advanced Tumors

A cohort of 17 patients was treated with cemiplimab for locally advanced CSCC. Invariably, these patients were referred by surgeons after multidisciplinary evaluation, as surgery was deemed unlikely to encompass all diseases, tumors were in anatomically sensitive sites, or had relapsed after prior surgery and/or RT. Some patients with locally advanced tumors had previously declined surgical management. Potential follow-up was a median of 30.5 months (range from 3.7 to 49 months). A total of 12/17 (70.6%) patients achieved a CR, two patients had a PR (11.8%), and two patients had SD (11.8%). Only one of the patients with a locally advanced tumor developed a progressive disease (5.8%). Median progression free survival was 43.9 months (Figure 2). At the end of data collection, 11/17 (64.7%) of patients remained in an ongoing remission.

### 3.4. Cemiplimab for Treatment of Metastatic CSCC

Our series included 10 patients who presented with metastatic CSCC. Potential follow-up was a median of 32.9 ± 14.8 months (range of 6.6–53.8 months). Eight of these ten (80%) patients achieved a CR with cemiplimab treatment; two patients had a PR. Median progression-free survival was not reached in this subset (Figure 2). By the analysis date, all eight CR patients remained in a long-term complete remission. Both PR patients (20%) developed recurrent disease.

### 3.5. Cemiplimab Treatment of Innumerable CSCC

Nine patients, with no known causes of immunosuppression, had TNTC CSCC. Potential follow-up of the TNTC subset was a median of 42.8 ± 20.9 months (range of 5–60.9 months). Of these nine patients, two patients achieved an initial CR, six patients had a significant reduction in the number of lesions, one patient had SD, and zero patients had PD. The median progression interval (time to new lesions) was 18.9 months (Figure 2). By the end of the analysis period, only two patients (22.2%) with TNTC lesions remained free of new skin cancers, while 77.8% (7) patients had developed new CSCC lesions.

### 3.6. Cemiplimab Therapy with Concurrent RT

**Figure 3:**
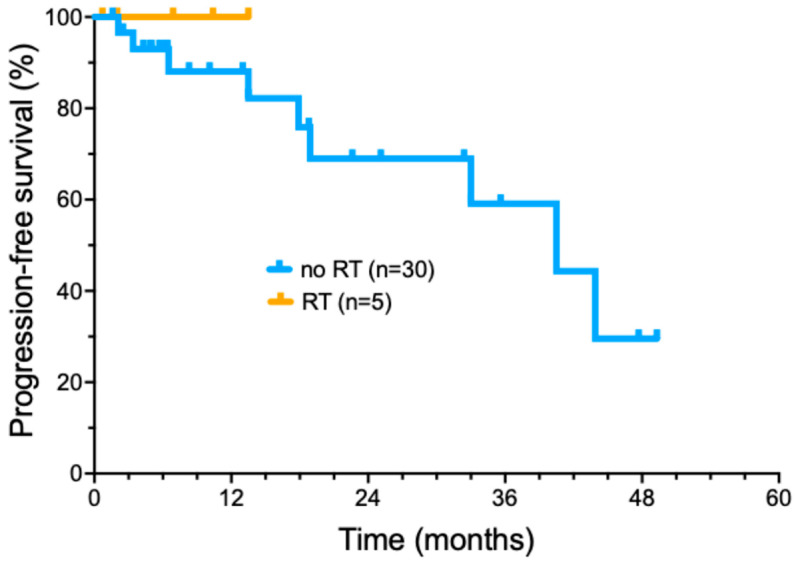
Progression-free survival of cemiplimab-treated cutaneous squamous cell carcinoma patients treated with radiotherapy added to cemiplimab treatment.

More recently, we have selectively added radiotherapy to cemiplimab treatment in patients who did not achieve a significant tumor response following induction cemiplimab therapy (four doses) (Figure 3). Five patients were treated with the addition of concurrent RT during ongoing cemiplimab treatment (Supplemental Table S3). A total of three out of five patients had locally advanced tumors, while two had regionally advanced metastatic disease. The delivered RT dose ranged from 6000 to 7040 cGy (median of 7000 cGy) delivered in 32–35 fractions (median of 32 fractions) delivered over 36–96 days. No patients had interruptions in RT due to toxicity. Cemiplimab with concurrent RT had toxicities that included bullous pemphigoid (1); moderate skin reaction and mucositis (1); and radiation dermatitis with pain and swelling of the treated area (1). All five patients treated with a combination of immunotherapy with involved field radiotherapy have remained in complete remission, with a short median duration of follow-up (16.2 months).

## 4. Discussion

Previously, treatment options for locally advanced or metastatic CSCC were quite limited due to lack of effectiveness and the increased age and frailty of this patient population. Surgery and radiotherapy were standard treatment options [1]. Chemotherapy and agents targeting the epidermal growth factor receptor (EGFR) demonstrated modest response rates [22,23,24,25,26,27]. Responses to chemotherapy and EGFR-targeted agents appeared to be quite transient. Chemotherapy proved to be poorly tolerated due to a significant level of toxicity. We previously showed that combination therapy with cetuximab and concurrent radiotherapy can produce durable responses in selected patients with locally advanced CSCC [28].

More recently, PD-1 antibody-based immunotherapy of CSCC has demonstrated significant antitumor activity. Some tumor-related factors observed in CSCC may contribute to the high response rates seen with immune checkpoint inhibitor (ICI) treatment. These include elevated levels of PD-L1 expression (a binding partner for PD-1) in CSCC [29]. In addition, a high tumor mutation burden in CSCC is believed to increase the expression of tumor-specific antigens, facilitating immune recognition and cytotoxicity [29].

Trials with PD-1 inhibitors have demonstrated significant clinical efficacy in patients with unresectable or metastatic CSCCs. For example, Maubec et al. performed a phase II study with pembrolizumab that found an overall response rate of 41% among patients, with nearly half of these responses sustained for 6 months or more [17]. In another phase II study, nivolumab achieved a 58.3% objective response rate, with some responses showing durability over extended follow-up (17.6 months) [30]. A third PD-1-directed monoclonal antibody, cemiplimab, has also demonstrated significant activity in CSCC patients, including those with locally advanced and metastatic disease [16]. In this phase II study, a 50% RECIST response rate (based on radiologic assessment or clinical photography) was observed following cemiplimab in patients with a locally advanced disease. Similarly, a response rate of 47% was observed in a metastatic-disease cohort. Durable responses were observed, although the published median follow-up of this trial was relatively short (8.7 months) [16]. In a subsequent confirmatory open-label study of cemiplimab, a significant response percentage was confirmed, with a median progression-free survival of 26 months [31].

One of the problems of clinical trials is that these patients are frequently highly selected based on study entry criteria, as well as superior performance status. Thus, clinical trial outcomes may not mirror what is seen in clinical practice. In a real-world evaluation by Kuzmanovski et al., 25 patients were treated with cemiplimab. These investigators observed an objective response rate of 52% (three complete and ten partial responses) [32]. Toxicity appeared to be significant in this trial, with a 36% rate of serious adverse events. In fact, six patients (24%) were withdrawn from treatment due to toxicity.

Verkerk et al. published data from 151 patients enrolled in a registry trial in the Netherlands [33]. The physician-assessed objective response rate was 35.1%. With a median follow-up of 15.2 months, median progression-free survival was only 12.2%. However, serious adverse events occurred in 29.8% of their patients.

Another 2021 phase II study by Rischin et al. of 193 patients with locally advanced or metastatic CSCC showed a median follow-up of 15.7 months. The overall objective response rate was 46.1%. The Kaplan–Meier estimated 24-month overall survival rate was 73.3%. Improvements in quality of life were evident with lasting and significant benefits through the final assessment. This study reinforced our findings, confirming the sustained clinical activity of cemiplimab, including increasing CR rates and longer response durations [34].

Similarly, in the phase II study by Migden et al., 78 patients with locally advanced CSCC were evaluated. The objective response rate was 44%, with 10 patients (13%) achieving a CR and 24 patients (31%) showing a PR. Moreover, 44% of patients experienced grade 3–4 treatment-emergent adverse effects, with hypertension being the most common, affecting six patients. Serious adverse events were reported in 29% of patients, including one treatment-related death [18].

Our findings confirmed a high level of activity of cemiplimab in locally advanced or metastatic CSCC. The onset of response was generally rapid and could frequently be detected after even one or two doses of cemiplimab. A 2021 review highlighted that retrospective studies on real-world responses to immunotherapy provide valuable insights into patients typically excluded from clinical trials, such as solid organ transplant recipients and those with autoimmune conditions [35]. The objective response rates in these real-world settings, ranging from 31.5% to 58.7%, were found to be similar to those in clinical trials. However, differing from these findings on objective response rates, here we find a higher complete response rate than previously reported (70–80%). In clinical trials, response assessment typically relied on imaging criteria (e.g., iRECIST) or clinical photographs to evaluate tumor shrinkage or stability [36]. In CSCC immunotherapy trials, residual scar tissue at a treated site may mimic persistent disease. This applies both to locally advanced tumors and to metastatic disease patients with stable radiographic residual lesions. In our experience, it is important to verify the degree of cancer response via a biopsy. This ensures that persistent stable abnormalities on clinical exams or imaging are not misinterpreted as active diseases. This may prevent unnecessary treatment continuation or escalation. Alternatively, if persistent tumor is identified, premature treatment discontinuation can be avoided. Most of our complete responses have proven durable, allowing eventual elective treatment discontinuation. We have previously published an effective strategy of treatment discontinuation for patients based on confirmation of a complete remission [21]. The benefit of continuing cemiplimab therapy beyond a CR may not outweigh the risks of long-term toxicity [37].

While the initial treatment response in patients with innumerable (TNTC) CSCC primaries was high, most of these patients (77.8%) eventually developed new CSCC lesions. It is not clear whether more prolonged treatment of these patients would be more effective. Thus, additional treatment strategies to prevent the development of new primary CSCC are needed for this patient subset.

We also performed an exploratory analysis of the potential benefit of adding radiotherapy for patients with minimal clinical response to four doses of cemiplimab therapy. All five patients who received concurrent RT added to ongoing cemiplimab therapy achieved a durable CR. This approach did not lead to any unexpected toxicities. However, it is important to note that CSCC is a radiosensitive tumor; the data do not necessarily demonstrate that the response was improved by the addition of cemiplimab. These patients might have achieved similar outcomes with RT alone. Thus, further prospective evaluation of this approach is warranted to determine whether the combination provides additional benefit over RT alone and to confirm the observed high level of local control in locally advanced CSCC.

Our data also suggest that CSCC treatment with cemiplimab has manageable immunologic adverse events. Toxicities in our patients were similar to those observed in other trials of PD-1/PD-L1 blocking agents [38]. Fatigue appeared to be the most common side effect. A small percentage of patients experienced pruritus, rash, epigastric discomfort, fevers/chills, dizziness, balance issues, mild headache, hives, and significant cough. No patients were hospitalized due to treatment toxicity. Only one patient died of progressive CSCC following cemiplimab treatment. Two elderly patients died of non-cancer-related causes.

Our study provides real-world data involving diverse populations that may not be adequately represented in clinical trials and provides novel insights into cemiplimab effectiveness in recurring clinical scenarios. The strengths of the current study include consistent treatment by a single physician experienced in managing ICI toxicity. In addition, we report a relatively lengthy follow-up of treated patients.

Limitations of this study included the use of retrospective data acquired over a number of years. Thus, referral bias and other potentially confounding variables are hard to evaluate. Additionally, five patients were lost to follow-up, and two patients died of other age-related causes. Thus, due to the relatively small number of patients, our experimental results should be considered hypothesis-generating. Additionally, our study did not specifically address CSCC in additional important clinical subsets of CSCC patients, such as in the setting of lymphoproliferative disease, immunosuppression, or in transplant recipients.

## 5. Conclusions

We have confirmed a high level of clinical activity of cemiplimab in advanced CSCC patients treated in a community practice. Our study included a significant percentage of patients who achieved complete remissions and were able to discontinue therapy after radiologic or pathologic confirmation of complete remission. Patient subsets with locally advanced or metastatic CSCC frequently achieved lengthy remissions. We saw enhanced treatment activity following the addition of radiotherapy in patients who were not responding to cemiplimab after the initial four doses. While cemiplimab treatment proved active in reducing the number of existing lesions in patients with TNTC CSCC, there is a need for novel approaches to reduce the high risk of subsequent new primary skin cancer development in this patient subset.

## Data Availability

De-identified primary data will be made available upon reasonable request to the corresponding author.

## Supplementary Materials

The following supporting information can be downloaded at: https://www.mdpi.com/article/10.3390/cancers17050823/s1. Table S1: Patient characteristics and treatments. Table S2: Treatment outcomes. Table S3: Cemiplimab + RT.

## Abbreviations

The following abbreviations are used in this manuscript:

AJCC	American Joint Committee on Cancer
CLL	Chronic lymphocytic leukemia
CR	Complete response
CSCC	Cutaneous squamous cell carcinoma
CT	Computerized tomography
EGFR	Epidermal growth factor receptor
ICI	Immune checkpoint inhibitor
IRB	Institutional review board
MRI	Magnetic resonance imaging
NHL	Non-Hodgkin’s lymphoma
PD	Progressive disease
PET	Positron emission tomography
PFS	Progression-free survival
PR	Partial response
RT	Radiotherapy
SD	Stable disease
SEER	Surveillance, Epidemiology, and End Results
StDev	Standard deviation
TNTC	Too numerous to count
UICC	Union for International Cancer Control
UPN	Unique patient number
