# Supplementary material for: Effectiveness and Toxicity of Cemiplimab Therapy for Advanced Cutaneous Squamous Cell Skin Cancer in a Community Oncology Practice
Source: Cancers (Basel). 2025 Feb 27;17(5):823. doi: 10.3390/cancers17050823 (PMC11899135; doi:10.3390/cancers17050823)
Supplement: Supplementary file 1 [file cancers-17-00823-s001.zip › Supplemental Table 1.pdf]

Table S1
Patient characteristics and treatment

UPN	Age	Sex	Race	Description	Primary Site	Metastatic Site	Comorbidities	Cemiplimab doses	Cemiplimab duration (months)	Potential follow up (months)
1	68	M	C	M	Unk	LN	HTN, osteoarthritis	8	4.9	38
2	64	F	C	TNTC	multiple	None	Alcoholism, pancreatic atrophy	8	8.5	49
3	84	M	C	M	scalp	In-transit	Osteoarthritis, HTN, prostate CA, hypothyroidism	6	4.6	21.4
4	77	M	C	M	Unk	LN	Asthma, hypercholesteremia	8	5	29
5	84	M	C	LA	scalp	In-transit	CHF, T2DM, CRF neuropathy, HTN	11	9.1	11.6
6	80	M	C	M	face	Parotid	Arthritis, HTN, prior stroke, gout, hyperlipidemia, prostate CA	7	6.4	6.6
7	85	M	C	LA	Scalp	None	Arthritis, T2DM neuropathy, HTN, CRF, stroke, hypercholesteremia	4	2.8	57.3
8	57	M	C	M	Face	Parotid	None	10	6.6	14.3
9	67	F	C	M	face	Parotid	None	8	5.1	53.8
10	79	F	C	LA	Scalp	None	Asthma, HTN, intermittent bronchitis	10	6.7	14.3
11	72	M	C	LA	Scalp	Parotid	Arthritis, COPD, HTN, gout, BPH, low testosterone, Rai 0 CLL	8	7.8	23
12	81	M	C	LA	face	Bone	Prior CSCC	8	5.9	9.5
13	88	M	C	LA	Face	Parotid	None	8	5.3	54.5
14	82	M	C	LA	Scalp	None	Osteoarthritis, hypothyroidism, CAD, T2DM, HTN, spinal stenosis, bladder CA, hydronephrosis	12	7.7	61.1
15	54	F	C	LA	foot	None	Arthritis	2	1.6	8.4
16	49	M	C	LA	arms	None	HTN, COPD, GSW, alcoholism	4	3	52.1
17	77	M	C	M	face	In-transit	HTN, hypercholesteremia, indolent NHL	8	5	33.8
18	79	M	C	LA	scalp	None	HTN, Parkinsonism, dementia,	7	4.3	22.1
19	77	M	C	LA	arm	None	Treated hepatitis C	8	5.7	7.7
20	64	F	H	LA	face	None	HTN, T1DM, restless leg syndrome, arthritis, hypothyroidism	6	5.1	35
21	77	M	C	M	face	In-transit	Arthritis, HTN, GERD	8	5.2	35
22	62	M	C	LA	face	None	None	2	0.7	19.6
23	66	M	C	LA	face	None	HIV, MI	20	17.1	48
24	61	F	C	TNTC	multiple	None	None	5	3.6	9.5
25	75	F	C	TNTC	multiple	None	COPD, HTN, diverticulitis, GERD, depression	2	0.9	42.8
26	84	M	C	TNTC	multiple	None	AF, pacemaker, cholecystectomy	8	6.2	19.1
27	60	M	C	LA	arm	None	Bipolar illness, arthritis, sarcoidosis, testicular torsion	4	2	3.7
28	87	M	C	TNTC	multiple	None	AF, pacemaker, arthritis	3	1.8	52.9

29	72	M	C	M	Unk	LN	BPH, hypogonadism, indolent NHL	8	4.8	49.8
30	76	M	C	M	Parotid	Parotid	Crohn’s disease	8	5.5	32
31	80	M	C	LA	Ear	None	Stroke, HTN, gout	8	4.8	43
32	63	F	C	TNTC	multiple	None	T2DM, HTN, hypothyroidism, hypercholesteremia	10	7.4	54.3
33	82	F	C	TNTC	multiple	None	Arthritis, HTN, AF, hypercholesterolemia	8	5.4	60.9
34	61	M	C	LA	Scalp	None	Psoriasis	39	33.9	35.4
35	83	M	C	TNTC	multiple	None	Arthritis, BPH, hyperglycemia, HTN	4	2.2	5
36	56	F	C	TNTC	multiple	None	None	4	6.4	27.3

Abbreviations: UPN, unique patient number; M, male; F, female; C, Caucasian; H, Hispanic; M, metastatic; LA, locally advanced, TNTC, too numerous to count primary cutaneous tumors; Unk, unknown primary site; LN, Lymph node metastases; TNTC, too numerous to count; HTN, hypertension; CHF, congestive heart failure; T2DM, type 2 diabetes mellitus; CRF, chronic renal failure; BPH, benign prostatic hypertrophy, CLL, chronic lymphocytic leukemia; CSCC, cutaneous squamous cell carcinoma; CAD, coronary artery disease; GSW, gunshot wound; NHL, non-Hodgkin’s lymphoma; T1DM, type 1 diabetes mellitus; GER, gastroesophageal reflux disease; HIV, human immunodeficiency virus; MI, myocardial infarction; COPD, chronic obstructive pulmonary disease; AF atrial fibrillation.
