# Supplementary material for: Effectiveness and Toxicity of Cemiplimab Therapy for Advanced Cutaneous Squamous Cell Skin Cancer in a Community Oncology Practice
Source: Cancers (Basel). 2025 Feb 27;17(5):823. doi: 10.3390/cancers17050823 (PMC11899135; doi:10.3390/cancers17050823)
Supplement: Supplementary file 1 [file cancers-17-00823-s001.zip › Supplemental Table 2 rev.pdf]

Table S2
Treatment outcome

UPN	Description (LA stage)	Cemiplimab Toxicity	Radiotherapy Toxicity	OR	PFS (months)	OS (months)	Current Status	Cause of Death
1	M	None	–	CR	35.6	35.6	NED	–
2	TNTC	Generalized pruritic rash, erythema at tumor sites, epigastric pain	–	CR	13	38.8	NED	–
3	M	None	–	CR	18.8	18.8	NED	–
4	M	Fever/chills, rash (mildly pruritic), dizziness	–	CR	25.1	25.1	NED	–
5	LA-T4B	Bullous pemphigoid	None	CR	10.4	10.4	NED	–
6	M	None	–	PR	6.4	6.4	AWD	–
7	LA-T4B	None	–	PD	2.1	6.6	Died-other	Subdural hematoma
8	M	None	Dermatitis & mucositis	CR	13.5	13.5	NED	–
9	M	None	–	CR	49.9	49.9	NED	–
10	LA-T3	None	–	CR	13.5	13.5	NED	–
11	LA-T3	Balance issues, muscle weakness, dizziness, asthenia	–	CR	22.6	22.6	NED	–
12	LA-T4b	Skin rash	No RT toxicity	CR	6.9	6.9	NED	–
13	LA-T3, parotid invasion	None	–	CR	40.5	40.5	NED	–
14	LA-T4b	None	–	CR	43.9	59.6	DOD	PD
15	LA-T3	None	–	SD	1.6	1.6	AWD	
16	LA-T3	None	–	CR	47.7	47.7	NED	-
17	M	None	–	PR	5	5	AWD	–
18	LA-T3	One episode of diarrhea, currently has loose stools	–	PR	4.3	5	Died-other	COVID 19 pneumonia
19	LA-T3	None	–	CR	5.7	5.7	NED	–
20	LA-T4B	Pain in tumor, anxiety, watery diarrhea with cramps, mild headache	–	CR	32.4	32.4	NED	–
21	M	Lightheadedness, arthralgias, fatigue	None	CR	0.7	32.4	NED	–
22	LA-T3	None	–	PR	33	0.7	AWD	–
23	LA-T3	None	–	CR	33	33	NED	–
24	TNTC	Rash, hives, SOB, significant cough	–	PR	8.3	8.3	AWD	–
25	TNTC	Lichen planus on chest and back	–	SD	3.4	3.4	AWD	–
26	TNTC	None	–	PR	6.2	6.2	AWD	–
27	LA-T3	None	Dermatitis	CR	2	2	NED	–
28	TNTC	Increased fatigue, onset of significant diarrhea	–	PR	2.5	2.5	AWD	–
29	M	None	–	CR	49.3	49.3	NED	–
30	M	None	–	CR	10.1	10.1	NED	–
31	LA-T3	None	–	CR	17.9	40.6	NED	–
32	TNTC	Prurigo nodularis	–	CR	18.9	18.9	NED	–
33	TNTC	None	–	PR	6.5	6.1	AWD	–
34	LA-T3	Diarrhea	–	SD	13.5	33.9	AWD	–
35	TNTC	Rash, mild pruritus	–	PR	4.9	4.9	AWD	–

36	TNTC	Inflammation, crusting	–	PR	6.4	6.4	AWD	–

Abbreviations: UPN, unique patient number; M, metastatic; LA, locally advanced (TNM stage shown);TNTC, too numerous to count primary skin cancers; CR, complete response; PR, partial response; SD, stable disease; PD, progressive disease; PFS, progression free survival; OS, overall survival; NED, no evidence of disease; D-other, died of non-cancer related causes; DOD, died of CSCC
