# Supplementary material for: Effectiveness and Toxicity of Cemiplimab Therapy for Advanced Cutaneous Squamous Cell Skin Cancer in a Community Oncology Practice
Source: Cancers (Basel). 2025 Feb 27;17(5):823. doi: 10.3390/cancers17050823 (PMC11899135; doi:10.3390/cancers17050823)
Supplement: Supplementary file 1 [file cancers-17-00823-s001.zip › Supplemental Table 3.pdf]

Table S3
Cemiplimab + RT

UPN	Age	Sex	Tumor characteristic	Cemiplimab doses	Cemiplimab duration (months)	Radiotherapy modality	Total RT Dose (cGy)	RT fractions	Elapsed time (days)
5	84	M	Locally advanced	11	9.1	P	70.4	32	36
8	57	M	Regional	10	6.6	P	6600	33	47
12	81	M	Locally advanced	8	5.9	EB	70.4	32	49
21	77	M	Regional	8	5.2	P	6000	30	96
27	60	M	Locally advanced	4	2	EB	7000	35	50

Abbreviations: UPN, unique patient number; M, male; F, female; EB, electron beam radiotherapy ; P, photon radiotherapy
